# Editorial Comment: Diagnostic performance of MRI and US in suspicion of penile fracture

**DOI:** 10.1590/S1677-5538.IBJU.2023.03.03

**Published:** 2023-04-15

**Authors:** Luciano A. Favorito

**Affiliations:** 1 Unidade de Pesquisa Urogenital Universidade do Estado do Rio de Janeiro Rio de Janeiro RJ Brasil Unidade de Pesquisa Urogenital - Universidade do Estado do Rio de Janeiro - Uerj, Rio de Janeiro, RJ, Brasil

Paul Spiesecke ^1^, Josef Mang ^2^, Thomas Fischer ^1^, Bernd Hamm ^1^, Markus H Lerchbaumer ^1^*Transl Androl Urol. 2022 Mar;11(3):377-385.*

## COMMENT

Penile fracture(PF) is a type of penile trauma that requires emergency intervention (1-3). PF is defined as the rupture of the tunica albuginea (TA) of the corpora cavernosa (CC) caused by blunt trauma to the erect pênis. In most cases, that occurs during sexual relations, when the penis slips out of the vagina and strikes against the symphysis pubis or perineum and is more likely when the partner is on top (4-6).

The diagnosis of penile fracture is mainly clinical, made from a thorough history and physical exam alone (7-9). The patient often reports blunt trauma during intercourse accompanied by an audible “snap” or “pop,” followed by immediate pain and rapid detumescence. Physical exam findings may include edema, ecchymosis, and penile deformity, classically described as an “eggplant deformity” (1).

In the present paper the authors studied the further evidence concerning the diagnostic accuracies of magnetic resonance imaging (MRI) and ultrasound (US) in the diagnostic assessment of patients with suspected PF and concluded that the results of this study suggest that MRI is more suitable to confirm PF and identify the site of the associated tunica albuginea tear while US is a good tool for ruling out PF.
